# “Reasonable adjustments” under the UK's *Equality Act 2010*: An enquiry into the care and treatment to patients with intellectual disabilities in acute hospital settings

**DOI:** 10.1111/jar.12623

**Published:** 2019-06-20

**Authors:** Marcus Redley, Isabella Lancaster, Adam Pitt, Anthony Holland, Angela Thompson, John R. Bradley, Gyles Glover, Karen Thomson, Sara Jones, Bernadette Herbert, Anita Holme, Isabel C. H. Clare

**Affiliations:** ^1^ Department of Psychiatry University of Cambridge Cambridge UK; ^2^ School of Health Sciences University of East Anglia Norwich UK; ^3^ NIHR CLAHRC East of England Cambridge UK; ^4^ East and North Hertfordshire NHS Trust Cambridge UK; ^5^ Cambridge University NHS Health Foundation Trust Cambridge UK; ^6^ Public Health England Cambridge UK; ^7^ Cambridgeshire & Peterborough NHS Foundation Trust Cambridge UK

## Abstract

**Objectives:**

To understand the views of qualified medical practitioners regarding “reasonable adjustments” and the quality of the care and treatment provided to adults with intellectual disabilities when admitted to acute hospitals as inpatients.

**Methods:**

Semi‐structured interviews took place with 14 medical practitioners, seven from each of two acute hospitals, with a thematic analysis of the resulting data.

**Results:**

All 14 medical practitioners reported problems in the diagnosis and treatment of patients with intellectual disabilities. Most participants attributed these difficulties to communication problems and/or behaviours that, in the context of a hospital ward, were non‐conforming. However, a minority reported that, because they were likely to have multiple comorbid health conditions, patients with intellectual disabilities were more complex. In addition, half of all these respondents reported making little use of “reasonable adjustments” introduced to improve the quality of the care received by this group of patients.

**Conclusions:**

Medical practitioners should make better use of the “reasonable adjustments” introduced in the UK to address inequities in care and treatment received by patients with intellectual disabilities. However, training should also focus on the biomedical complexities often presented by these men and women.

## 1 INTRODUCTION

Globally, health care for men and women with intellectual disabilities receives very limited or no attention during medical training (Salvador‐Carulla & Saxena, 2009) and is an area where a large gap exists between the health needs of this population and the provision of services (World Health Organization, 2007). Seeking to address such inequalities, Article 25 of the UN's *Convention on the Rights of Persons with Disabilities* (UN, 2006) requires States Parties to recognize that persons with disabilities have the right to enjoyment of the highest attainable standard of health without discrimination.

Even in an economically well‐resourced state such as the United Kingdom, however, people with intellectual disabilities experience poorer quality health care than their non‐disabled peers, leading to avoidable premature deaths (Heslop et al., 2014). Some of these deaths take place in acute hospitals, where it has been alleged that patients with intellectual disabilities experience “institutional discrimination” (p. 2, Mencap, 2007). Of concern, Heslop and her colleagues' data were collected after the introduction of the *Equality Act* 2010. The *Equality Act*, which replaces previous disability discrimination legislation, specifies that all public services, including acute hospitals, are required to make “reasonable adjustments” to ensure that people with disabilities, including those with intellectual disabilities, are not “substantially disadvantaged” (*Equality Act* s.20: 1–5).

A survey of “reasonable adjustments” in acute hospitals (Hatton, Roberts, & Baines, 2011) noted the introduction of a range of measures for patients with intellectual disabilities. These included the provision of specialist nurses (“learning disability”) and other liaison nurses and “passports” detailing individuals' support and communication needs. At the same time, autonomous decision making by patients with intellectual disabilities was supported by the provision of easier‐read information, while staff received training in the *Mental Capacity (England & Wales) Act* 2005. The *MCA* both promotes decision making by adults with “an impairment of, or disturbance in the functioning of, the mind or brain” (s.2(1)) and regulates substitute decision making for those individuals judged to lack capacity to make for themselves one or more decisions about their care and treatment. Hatton et al. (2011), however, comment that insufficient data are available regarding the effectiveness of these measures. Nevertheless, there have been a number of small‐scale and largely qualitative studies investigating the effectiveness of efforts to improve the quality of the care received by patients with intellectual disabilities: the findings have not been encouraging. For example, Atkinson (2016), using self‐reports from fifteen nurses, found that, even when they were available, patients' hospital passports seemed not to be used. In a larger study, Northway and her colleagues (Northway, Rees, Davies, & Williams, 2017) examined 60 passports developed by healthcare provider trusts across the UK. Key information relating to, for example, allergies, risk assessments and the need for reasonable adjustments, was either not included or difficult to locate. Furthermore, a review of 14 research papers relating to the experiences of nurses in acute settings suggested that these practitioners felt unprepared for caring for people with intellectual disabilities, found it hard to communicate with them, and were uncertain about the support they might expect from paid and family caregivers (Lewis, Gaffney, & Wilson, 2017).

Despite many initiatives since Mencap's (2007) ground‐breaking report in the UK, patients with intellectual disabilities continue to have poor experiences during admissions to acute hospitals (Iacono, Bigby, Unsworth, Douglas, & Fitzpatrick, 2014).

Much of the research into the care and treatment received by patients with intellectual disabilities in acute hospitals is published in nursing journals and is focused on the work and/or experiences of nurses. This creates the impression that of all the healthcare practitioners working in acute hospitals, it is nurses who are primarily responsible for improving the care and treatment received by patients with intellectual disabilities. In an attempt to engage critically with this orthodoxy, we sought to introduce another voice, that of medically qualified doctors working in acute hospitals, hereafter referred to as “medical practitioners.”

Medical practitioners have a lead role in the care and treatment received by all patients. Through their responsibility for explaining patients' symptoms by taking oral histories and carrying out physical examinations, and investigations, they aim to arrive at a list of possible diagnoses (differential diagnoses) and ultimately a final diagnosis that will inform the intervention (Peterson, Holbrook, Von Hales, Smith, & Staker, 1992). To carry out these tasks, medical practitioners are dependent upon patients being able to provide an account of their symptoms, recall their medical history and, if needed, give consent for investigations, which in some cases may be invasive, distressing and not without risk. As such, the views and experiences of medical practitioners may help to further our understanding of how best to ensure that patients with intellectual disabilities receive equitable care and treatment.

## 2 METHOD

Following ethical approval from the National Research Ethics Service (14/WA/0148), semi‐structured interviews were conducted with a sample of medical practitioners at two acute hospitals in the UK. The interviews were designed to elicit participants' views about, and experiences of: (a) the care and treatment of patients with intellectual disabilities; (b) “reasonable adjustments” to improve the quality of care and treatment; and (c) working with family members and paid social caregivers. In addition, (d) participants were asked to consider whether patients with intellectual disabilities might receive poorer quality care and treatment than their non‐disabled peers.

Medical practitioners were considered eligible for inclusion in the study if: (a) they had been involved in the care and treatment of a patient identified as having an intellectual disability *and* (b) consent for us to invite them to participate had been given by the patient themselves or we had received favourable advice from a family carer of someone judged to lack capacity to give or withhold consent to taking part (s. 30 ff., *MCA*). Consent/favourable advice was obtained from thirty patients, relating to 40 eligible participants (some patients had been cared for by more than one medical practitioner). Consent was then sought from the potential participants. Eventually, interviews were conducted with 14 participating medical practitioners, seven from each hospital. This was a convenience sample comprising: three specialists in each of renal medicine and acute care; two in each of the following specialisms, surgery, neurology and respiratory medicine; and one each in, respectively, trauma and hepatology. The duration of the interviews varied, ranging from 15 to 60 min, as we needed to accommodate demands of the participants' clinical work. It should be noted that, while medical practitioners were recruited through their association with a specific patient, they were not specifically asked to comment on that person's care and treatment since it was not our intention to compare the views of the medical practitioner–patient dyad. Nor was any attempt made to examine differences between the respondents in the two hospitals.All interviews were conducted face‐to‐face and audio‐recorded. As we wanted to understand why patients with intellectual disabilities might receive poor quality care, the interviewers were encouraged to adopt a challenging stance in order to develop a lively discussion so that medical practitioners might be called upon to defend their practice (Holstein & Gubrium, 2016).

The interviews were transcribed verbatim. Since these were semi‐structured interviews, each interview question corresponded with a different interpretative theme (Cicourel, 1964). Participants' responses were, consequently, coded question by question, using NVivo, and then summarized. These summaries were refined and validated through meetings of the research team. Our analysis takes participants' accounts at face value (Silverman, 2017); no attempt is made to explore how respondents' rhetorical construction might justify their practice (Wetherell & Potter, 1988). Consequently, the quotations presented serve purely to illustrate the kinds of observations participants made.

## 3 RESULTS

### 3.1 Introduction

When asked about the relevance of patients' intellectual disabilities to the provision of care and treatment, all the medical practitioners reported that it was highly significant, with both diagnosis and treatment being perceived as “challenging.” The majority of respondents made much of the difficulties they experienced in communicating with, and managing what was perceived as the non‐conforming behaviour of patients with intellectual disabilities. In contrast, a minority focused primarily on the biomedical complexities of this patient group. When the challenges of treating patients with intellectual disabilities were formulated in terms of communication, medical practitioners reported that it was difficult to obtain accounts of any current pain or discomfort, and even harder to construct the history of experiences of the symptoms (see Box 1: Excerpt 1). In addition, they described difficulties in providing care and treatment for patients who were judged to lack decision‐making capacity and/or whose non‐conforming behaviour, such as shouting or walking about, was viewed as likely to disrupt the smooth running of a ward. When the challenges of treating patients with an intellectual disability were formulated in terms of their biomedical complexity, medical practitioners referred to the presence of multiple comorbid health conditions; the prevalence of polypharmacy, particularly with regard to anticonvulsant medication for epilepsy; and the prevalence of neurodevelopmental syndromes with a genetic origin (see Box1: Excerpt 2). These two narratives, while not mutually exclusive, because some participants referred to both, nevertheless presented different ways of understanding the significance of a patient's intellectual disability, each with specific implications for addressing inequalities in health care. We begin this account of our findings by reviewing the dominant narrative, before describing medical practitioners' reported use of “reasonable adjustments,” and their responses to the suggestion that patients with intellectual disabilities may receive poorer quality care. Finally, we turn to descriptions of the impact of their reported biomedical complexity on treating patients with an intellectual disability.

Box 1 **Excerpt 1**

[Patients with intellectual disabilities] may not be able to verbalise their symptoms like you and I might do. […] So, to give you an example, they may just become very agitated and restless and not be able to tell me that they're in urinary retention or they've got constipation or something very simple, which an average adult who I can communicate with very easily would be able to tell me. [Acute Medicine: 1 of 3]

**Excerpt 2**

For example a patient with Down Syndrome we treated [for a kidney condition], I think it's recognising that because of the complexities of cardiac problems that Down Syndrome patients get [pause] their life expectancy may be different and actually understanding that and knowing that they've got potential cardiac problems because they've got Down Syndrome, is all part and parcel of the syndrome. [Renal Medicine: 1 of 3]

### 3.2 Difficulties in managing communication problems and non‐conforming behaviours

When describing their responses to the communication and behavioural challenges presented by patients with intellectual disabilities, medical practitioners reported a number of strategies. These included trying to spend more time with the patient, simplifying the complexity of their language, and reducing the number of investigations such as blood tests and scans that might cause distress (Box 2: Excerpt 1) and the use of proxies. Proxies, particularly family members, were described as invaluable because they were perceived as being able to give information about a patient's symptoms and medical history; provide a biomedical benchmark against which treatment goals could be set and evaluated by describing the patient in optimal health; facilitate communication between hospital staff and the patient; and manage the patient's anxieties (Box 2: Excerpt 2) that could, in extreme cases lead to non‐conforming behaviours, such as removing cannulas or distressing other patients. While making little distinction between family members and paid caregivers, since both were seen as holders of details of a patient's symptoms and medical history, some of the medical practitioners asserted the need to get families, in particular, “on board.” By this, they appeared to mean being sensitive to a family's concerns about the patient's health and listening to accounts of pervious hospital admissions that had been particularly distressing for the patient and/or their family member. Equally, however, participants reported that it could also mean explaining to family members that the level of personalised support available at home could not be reproduced in hospital. Further, it was reported that the need to involve families, paid caregivers and other relevant persons, such as advocates, could delay the commencement of treatment.

Box 2 **Excerpt 1**

I just talk to them [patients with intellectual disabilities] and you work out what their level and what kind of language and analogies to use and then you just go from there. Everyone's different. There's no specific strategy I use. Over the years, everyone develops their own set way of talking to people in terms of how they phrase things and I have my own set ways, but you adapt it for the patient. [Acute Medicine: 2 of 3].

**Excerpt 2**

Many patients with learning difficulties may feel unsettled in a strange environment and they get confused and agitated with a strange place around them. So, having the same person who normally looks after them gives them some comfort and confidence and facilitates treatment overall. [Renal Medicine: 2 of 3]

### 3.3 Reasonable adjustments

Only half of the 14 participants recalled working with a liaison nurse, such as a "learning disability" nurse and even, among those medical practitioners who had done so, knowledge of this specialist nurse's involvement could be vague (see Box 3 Excerpt 1). That said, three medical practitioners did give accounts where a “learning disability” liaison nurse had supported a patient and their family by alleviating anxieties about a complex investigation (MRI scanning), provided a sense of continuity at a time when patients and their family member are meeting a bewildering variety of clinical staff, and given useful assistance when making complex clinical decisions (see Box 3: Excerpt 2). However, respondents also reported that the information provided by the “learning disability” liaison nurse was no fuller than that provided by patients' caregivers. As for patient “passports,” which just over half of our sample reported having seen, participants' views were again sharply divided. While passports were viewed by some as a useful source of information about, for example, patients' expressive and receptive communication skills and support needs, others reported that they were often unnecessarily, and impracticably long, or raised doubts about the accuracy of the information they contained (see Box 3; Excerpt 3). While the medical practitioners were aware of other kinds of “reasonable adjustments” such as “flags” alerting staff to a patient's intellectual disability, easier‐read documentation about health conditions and medication, and specialist communication support, these were mentioned only in passing.

Box 3 **Excerpt 1**

Whether we do that routinely [involve the "learning disability" nurse], whether we could be better at doing that and getting them involved, I'm sure there's always room for improvement but I think we're reasonably good. Honestly, I'm not sure how [the "learning disability" nurse] would become involved. So, would it be that you would have to contact her or would a nurse be able to do that, would a healthcare assistant be able to do that? [Respiratory Medicine: 1 of 2]

**Excerpt 2**

I can't remember the details quite well enough to say exactly what her [the "learning disability" nurse] input was, but I do remember us having quite a reasonable conversation about how aggressively we should pursue treatment and she was someone who'd met that patient several times before so had quite a good grasp of the issue. [Acute Medicine: 3 of 3]

**Excerpt 3**

So sometimes the passports can be inaccurate and sometimes they're not brought with them. But yes, on the whole if they are brought with them, then actually simple things like what they like to be called or what they like to be fed or what they like to do, what they like to listen to music‐wise or just simply what they don't like, for instance, those sort of things can be very helpful. [Acute Medicine: 1 of 3]

### 3.4 Poorer quality care

When asked about family carers' complaints about having to provide the same basic information repeatedly (Michael, 2008), our medical practitioners were unapologetic. They explained that it was important for them to hear information first‐hand or from a proxy who knew the patient very well. Healthcare records, they reported, did not provide the detail, nor the immediacy, of a face‐to‐face exchange. They reported that interviews with the patient and/or their proxies provided valuable material relevant to their medical histories, allowed them to corroborate information from different sources and provided an insight into the care and treatment needs of particular patients (see Box 4: Excerpt 1). We gained the impression that repeated requests for the same information were regarded as inherent to care and treatment within acute hospitals and were not a particular feature associated with admissions of patients with intellectual disabilities.

When asked whether patients with intellectual disabilities were likely to receive care and treatment that was of a poorer quality than that of other patients, most participants agreed. Their responses drew on factors that characterize or are associated with an intellectual disability (communication difficulties [see Box 4: Excerpt 2]) and/or problems conforming to the “rules” of care and treatment in hospital. Importantly, those few participants who offered different accounts attributed their views to the introduction of “learning disability” liaison nurses, and, as a result, increased awareness among hospital staff of the needs of patients with intellectual disabilities (see Box 4: Excerpt 3). Two participants did not subscribe to either account. One suggested that staff shortages and increased workloads meant that “quieter” patients, including, contrary to other participants' views, those with intellectual disabilities, were at greater risk of neglect. The other participant's account focussed on the negative impact of “over‐zealous” campaigning on behalf of people with intellectual disabilities. Apparently, this led medical practitioners and other staff to be so fearful of “getting it wrong” that they sought to avoid these patients as much as possible. When asked specifically about why avoidable readmissions to hospital might be proportionately greater among patients with intellectual disabilities than their peers (Kelly et al., 2015), most participants attributed the finding to deficiencies in community services (see Box 4: Excerpt 4). It was reported that General Practitioners, and family and paid caregivers either failed to follow post‐discharge care plans, or if signs of ill health persisted, “played it safe” by referring patients back to hospital. There were, however, a small number of medical practitioners who reported that data relating to patients with intellectual disabilities could not reasonably be compared with that of their peers: in their view, those with intellectual disabilities were simply “more unwell” in that they had a much greater number of physical health comorbidities (see Box 4: Excerpt 5).

Box 4 **Excerpt 1**

Carers might think “why aren't you communicating, why haven't you asked the other doctor.” In fact, for me, I want to hear it in their own words. I'll have often scanned it [the notes], I'll see the story and I'll think, I know that's what's written and I actually want to hear what you say. I don't want what an inexperienced junior doctor has interpreted it as. I want to hear it from you […] and the things that worry you as the carer or the patient. [Neurology: 1 of 2]

**Excerpt 2**

I think it is probably largely around communication difficulties and understanding what the issues are for an individual […] I think it is largely around that. I don't think that there is any discrimination, if you like, not primary discrimination. [Neurology: 2 of 2]

**Excerpt 3**

I don't think they get a poor standard of care [patients with intellectual disabilities] but the Learning Disabilities Team can point out areas where the care can be optimised for that group of patients, I think. For example, getting them listed at the start of a list of procedures or letting the junior doctor team know that, if they need a cannula for intravenous fluid, that they would like some kind of support with them while you do that. Or whatever it might be, I'm sure that helps. But I don't think they get a poor standard [of care] as far as I'm concerned. [Hepatology: 1 of 1]

**Excerpt 4**

So, people [General Practitioners and paid caregivers] will often play it safe because they don't know the patient and because they can't communicate with the patient, they'll just go “I'll send him to hospital.” And that works for out‐of‐hours or you may have carers who don't know the patient. [Acute Medicine: 1 of 3]

**Excerpt 5**

Preventable readmission, that's an interesting one, in the sense that I'm not sure that they do [experience higher rates of readmission]. I think you're talking about a group of patients, if they come into hospital at all, are probably coming in with a certain amount of frailty and a potential for recurrent problems. The patient with cerebral palsy who's prone to chest infections, a patient with Down's dementia who's got an unsafe swallow and may aspirate. [Acute Medicine, 3 of 3]

### 3.5 Biomedical complexity

By emphasising the biomedical realities of the lives of some patients with intellectual disabilities, a minority of participants drew our attention to the complexities of providing treatment for individuals who might, in addition to their presenting healthcare need, be individuals with a neurodevelopmental syndrome associated with specific physical complications, be prescribed an idiosyncratic combination of medications, have extensive healthcare records and have comorbid health conditions (see Box 5: Excerpt 1). To illustrate this point, three participants provided brief accounts of the challenges they had experienced in providing health care to this biomedically complex group.

The first account was related to a woman with Down syndrome (Trisomy 21) who was diagnosed with pneumonia. The participant described feeling uncertain about whether the low oxygen levels in this patient's blood were attributable to the pneumonia or to the long‐term effects of Eisenmenger's syndrome, a congenital heart defects associated with Down syndrome. With no knowledge of the patient's “normal” blood‐oxygen level, the respondent felt unable to formulate an appropriate treatment. Reflecting their concern about the patient's low level of oxygen, nursing staff seemed insistent on giving supplementary oxygen; however, this is contra‐indicated in Eisenmenger's syndrome. In an example of the important role that caregivers can play, the medical practitioner's dilemma was resolved when the patient's family provided information about her optimal blood‐oxygen level obtained from the specialist outpatient clinic she attended at another hospital (see Box 5: Excerpt 1).

Box 5 **Excerpt 1**

Her oxygen levels got very, very low, how much of that was her Eisenmenger's, whether her Eisenmenger's was getting a lot worse or whether how much of that was attributed to what we thought was the infection in her chest? […] so clinically that's very challenging because you've always got that slight doubt or question in your mind which of the two is it, I'm dealing with? [Renal Medicine 3 of 3]

**Excerpt 2**

It became clear a couple of days down the line that actually he'd presented with a pneumonia […]and actually died a few days later after a lot of challenging conversations around how appropriate it was to escalate treatment or not, and it clearly wasn't and it wouldn't have been appropriate to take him to intensive care, for example. That was quite difficult to explain to relatives who were understandably both very upset that he was unwell, upset that we were late to make a correct diagnosis and felt quite strongly I think that we probably weren't doing everything we might have done in someone who didn't have a learning (intellectual) disability [Acute Medicine, 3 of 3]

**Excerpt 3**

The patient was just stuck in the middle of it [a decision that he be nil by mouth and have easier to swallow food] he couldn't contribute to that conversation at all […] Whereas if it was somebody who had no learning issues, they might have said, “Yes, but Dr Jones said yesterday that I wasn't to have anything by mouth until I'd finished having treatment for the pneumonia.” [Respiratory Medicine: 1 of 2]

The second account also concerns a person with Down syndrome: a man whose description of his symptoms initially led the treating medical practitioner to make an erroneous diagnosis. The patient's description of diarrhoea and vomiting was consistent with gastroenteritis. Following a rapid and serious deterioration in his health, however, it became clear that the correct diagnosis was that of pneumonia. The delay in diagnosing the patient correctly, which the participant attributed, in part, to the patient's difficulties in providing accurate information about his symptoms led to the clinician having to decide between admitting the patient to intensive care or withdrawing active treatment. In the medical practitioner's view, an intensive care admission would not prolong his life and would in all likelihood result in an unpleasant death. The decision was described as one that was very difficult to share with the man's family. Indeed, it led to the medical practitioner being the subject of a complaint and formal investigation (see Box 5: Excerpt 2).

Thirdly, and finally, we were told about a patient with an intellectual disability and Parkinson's disease who was admitted for recurrent aspiration pneumonia. The medical practitioner reported that it was unclear whether the patient's weak swallow was due to pneumonia, and so likely to improve with treatment, or was an irremediable consequence of the Parkinson's disease. While the patient had some spoken language, he was not able to convey whether his swallow had deteriorated. Aiming to minimize the risk of any further worsening of the patient's health, the respondent decided that while receiving antibiotics for the putative pneumonia, the patient should be *nil by mouth*. However, nursing staff, following advice from the speech and language team, started feeding the patient with pureed food. In concluding his account of this breakdown in communication, which could have endangered the patient's health, the participant observed that this situation could have been avoided if the person with an intellectual disability had been able to inform the nursing staff that he should not be eating (see Box 5: Excerpt 3).

These three accounts of the biomedical complexities of treating patients with an intellectual disability carry intimations of poor practice: a delay in diagnosis, resulting in pressure from nurses for the administration of inappropriate supplementary oxygen (account 1); an over‐reliance on the testimony of a person with an intellectual disability, with tragic consequences (account 2); and a failure in communication between the medical and nursing staff, potentially endangering the life of a very unwell patient (account 3). The accounts raise questions about the extent to which the implementation of s. 20 of the *Equality Act* might have ameliorated, or even avoided, challenging clinical situations.

## 4 DISCUSSION

This study of medical practitioners' views and experiences is limited by its small sample size, by the practicalities of carrying out interviews in a clinical setting and, more significantly, by the absence of complementary direct clinical observations that would support the interview data. Nevertheless, the findings reported here provide an opportunity to reflect upon the quality of the care and treatment that medical practitioners self‐report that they provide to patients identified as having intellectual disabilities.

Describing the challenges of providing care and treatment to patients with intellectual disabilities, medical practitioners focused on two accounts: the patients' communication difficulties and vulnerability to behaviours that did not conform to a hospital's expectations, and their biomedical complexities. Of these different accounts, the first was dominant; it represented what might be considered the orthodoxy established in the Michael Report (2008), with its focus on the importance of making “reasonable adjustments” consistent with equalities legislation. Similarly, studies of nurses working in acute settings (see review by Lewis et al., 2017) have reported overwhelmingly that communication and non‐conforming behaviours present the most complex challenges. Yet what was striking about our findings is that, while the majority of medical practitioners subscribed to this view, they reported making limited use of “reasonable adjustments.” Instead, they apparently turned to caregivers to facilitate communication and manage behaviours likely to upset hospital routines. With many family carers apparently remaining at the bedside throughout the admission of a person with an intellectual disability (Mencap, 2012), a certain reliance upon family care is perhaps understandable. However, while likely to satisfy family members' desire for involvement, there could also be some unintended adverse consequences. For example, this practice may, in part, contribute to the repeated requests by medical practitioners for the same information, about which family carers complain (Michael, 2008). At the same time, their constant presence may contribute to the evidence that, contrary to the *Mental Capacity Act*, medical practitioners (and other clinicians) believe that family members can make decisions on behalf of *any* adult identified as a person with an intellectual disability. Moreover, there is a danger that, by focusing on “reasonable adjustments” to minimize, the impact of reported challenges might eclipse no less significant biomedical complexities such as: comorbid health conditions (Cooper et al., 2015), polypharmacy (Haider, Ansari, Vaughan, Matters, & Emerson, 2014), and rare neuro developmental conditions (Redley, Pannebakker, & Holland, 2018). That the clinical needs of patients with intellectual disabilities are at risk of being overlooked should be of serious concern because they appear to be associated with sub‐optimal care and treatment. In keeping with the general neglect of people with intellectual disabilities during medical education and training (Salvador‐Carulla & Saxena, 2009), there is no recognized medical specialism relating to the care and treatment of this group of patients in acute settings. This contrasts strikingly with the situations of infants, children, and older people. Of concern, in response to questions about the high incidence of potential avoidable readmissions in this population, medical practitioners referred to deficiencies in the care being provided in community settings, rather than, as we would have hoped, reflecting on their own professional practice.

What action might be taken? Changes to medical education to include a much stronger focus on the *clinical* importance of compliance with equalities legislation and the relationship between the *Equality Act* and the *Mental Capacity Act* could go some way towards improving the situation. In this context, “reasonable adjustments” may assume greater relevance. Our recommendation that trainee medical practitioners in all specialisms should receive mandatory education and training in intellectual disability is hardly new: it was the first of the recommendations made by Michael (2008). Surprisingly, though material about the involvement of people with intellectual disabilities in medical education and training was already available (Owen, Butler, & Hollins, 2004), no guidance was provided about the curriculum or format that might be adopted. This is an area of work that requires further development. More broadly, the findings of this study were also consistent with an earlier analysis (Barnett et al., 2012), subsequently incorporated into the guidance about medical education and training produced by the UK's General Medical Council (2017). Both the analysis and the subsequent guidance emphasize the importance of generalist skills in responding to the increasing prevalence among patients of multi‐morbidities, often accompanied by polypharmacy. While the guidance was initially a response to the highlighted needs of an ageing population, patients with intellectual disabilities are also very likely to benefit from such an approach.

Notwithstanding the challenges that were identified by the respondents in this study, there remains the possibility that the care and treatment of patients with intellectual disabilities are related to more general problems in hospital care (Francis, 2013; Keogh, 2013). The observation by one respondent that “quiet patients” might be disadvantaged by staff shortages and increased workloads is consistent with research linking low levels of nurse staff to higher mortality rates (Rafferty et al., 2007). However, the relationship between staffing levels and mortality is complicated. There are variations between hospitals, wards and the medical needs of patients (National Institute for Health Research, 2019); substantiating any proposed link will not be easy. In the meantime, there is a need for observational and ethnographic studies to document in more detail the relationships between medical practitioners' self‐reported views and their actual care and treatment of patients with intellectual disabilities.
